# Using a six‐step co‐design model to develop and test a peer‐led web‐based resource (PLWR) to support informal carers of cancer patients

**DOI:** 10.1002/pon.4969

**Published:** 2019-01-16

**Authors:** Olinda Santin, Theresa McShane, Peter Hudson, Gillian Prue

**Affiliations:** ^1^ School of Nursing and Midwifery Queen's University Belfast, Medical Biology Centre Belfast UK; ^2^ The University of Melbourne, Centre for Palliative Care, St Vincent's Hospital Melbourne Australia

**Keywords:** cancer, caregiver, carer, information, intervention, oncology, resource, web‐based, website

## Abstract

**Objective:**

To co‐design and test the acceptability of a peer‐led web‐based resource (PLWR) for cancer carers to provide practical and emotional advice on common issues.

**Methods:**

A six‐step co‐design model informed PLWR development. Content was developed through three cancer carer workshops and monthly meetings with an expert advisory team (n = 12). User‐testing was conducted via web‐based survey and telephone interview. Descriptive statistics and thematic analysis were utilised. Google analytics explored site visits, commonly used components, and time spent using the PLWR.

**Results:**

The PLWR was developed to deliver cancer carer information tailored to each stage of the illness trajectory regardless of cancer type, in the form of videoed personal experiences. From November to May 2018, there were 2789 unique visits to the PLWR with 743 returners. The majority of time was spent on the full unclipped peer stories (414 views), and diagnosis‐specific information (159 views), with less time spent on bereavement, cancer treatment, or self‐care (120 views each). Fifty‐five individuals completed the resource evaluation, with 10 participating in telephone interviews. Fifty‐four carers rated the resource as excellent, useful, and easy to use. The web‐based videos were regarded as convenient as and less burdensome than written information. The resource provided relevant information, potentially reducing isolation and uncertainty.

**Conclusion:**

The content and design of the PLWR appear acceptable to cancer carers. The co‐design model is an effective way to develop appropriate information for service users and could be utilised as a framework for development of other interventions in a variety of disease groups.

## 1 BACKGROUND

A total of 1.5 million people aged 16 years and over in the United Kingdom (UK) are caring for someone affected by cancer.^1^ As the prevalence of cancer rises globally, so too does the number of people providing informal care. Informal carers can be individuals who look after family members, friends, neighbours, or others because of long‐term illness.^2^

Care provided by informal carers is vital given their assistance in managing medications, symptom management, personal care, and social support.^1^ Providing informal care can however have a negative impact on quality of life, physical, and mental health. Some carers report pain, poor sleep and fatigue,^3^ high levels of psychological difficulties,^4^, ^5^, ^6^ worry about a patient's health, and stress associated with providing care.^7^, ^8^, ^9^ Furthermore, the time and costs of providing care may lead to gaps in, or loss of, employment, reduced income, and overall financial strain.^1^

Poor carer health may compromise the ability to carry out their cancer caring role^3^, ^8^, ^10^ and is interdependent with patient outcomes,^11^, ^12^ particularly in relation to increasing patient distress and quality of life.^13^ Many cancer carers lack information about cancer and the expectations of their role, which can lead to worry and uncertainty.^14^ In addition, carers are rarely adequately prepared, as often the caring role is unexpectedly put upon them.^1^

In response to these needs, a number of online supports have been developed.^15^ These resources are not always developed from a strong evidence base, focus directly on the patient's needs, lack tailored information per stage of illness, focus predominantly in palliative care, and have had little input from patients and carers on their design.^14^ A growing body of research advocates that intervention development must bring together staff, patients, and carers to reflect on their experiences and work together to identify improvements.^16^, ^17^, ^18^ However, despite this recognition, the involvement of patients and carers in intervention design has been slow to develop.^16^, ^17^, ^18^

Despite the integration of routine patient information in oncology, specific guidelines and supports for carers are not routinely provided.^9^ Current health service provision is devoid of any specific statutory service for cancer carers, with the carer reliant on accessing services within the voluntary sector, and professionals feeling ill‐equipped to manage concerns.^9^

Interventions targeted at the cancer carer can improve outcomes such as quality of life and caregiver burden,^14^, ^15^, ^16^, ^17^, ^18^, ^19^ in particular those utilizing technology and a self‐directed format.^20^ A review of web‐based interventions for carers across various chronic illnesses found positive changes in psychological health, knowledge, and relationships; however, much of the quality of these studies were deemed as methodologically weak, and therefore more work on the subject is needed.^20^ It is argued that the best way to ensure that interventions meet the needs of the target population is to involve stakeholders in design and development.^21^, ^22^ This process known as co‐design has the potential to increase intervention effectiveness.^21^, ^22^

In response to rising carer pressures, the team co‐designed with cancer carers and health care professionals (HCP) a peer‐led web‐based resource (PLWR) to provide cancer carer specific practical and emotional advice on common carer issues. The acceptability of the resource was determined through user testing, examining carer's experiences of using the PLWR, and their ratings of the various components.

## 2 METHODS

The project was conducted in two phases: (1) a four‐step co‐design model to inform the development of the resource and (2) user testing evaluating resource acceptability (see Figure 1, Supporting Information). Development was guided by the MRC framework for developing complex interventions.^23^

### 2.1 Phase 1: Development of the PLWR

Early PLWR development was underpinned by a number of studies and a systematic literature review conducted by the authors identifying unmet needs in cancer carers.^9^, ^24^, ^25^ These studies^9^, ^24^, ^25^ clearly indicated a need to develop a resource by carers for carers that provided information to promote coping.^26^

#### 2.1.1 Step 1 PLWR design

To develop the PLWR content, cancer carers were asked to commit to three consecutive workshops at the regional cancer centre, the largest cancer unit in the region serving both urban and rural localities. Cancer carers who volunteered to participate in the workshop cared for patients of varying cancer sites and stages (n = 8). Carers were recruited via an email invite to the regional Northern Ireland Research Consumer Forum. This group includes carers and patients affected by cancer and ensures that the user voice informs research development. Each workshop was facilitated by OS and GP, recorded, transcribed, and thematically analysed (see Supplementary material). Through these workshops, a written draft of the PLWR structure and content was formed.

Following workshops, PLWR development was supported via six quarterly meetings with an expert advisory team. Advisory team meetings were facilitated and recorded by OS and TMcS. The expert team consisted of carers, academics, a men's health expert, cancer charity representatives, and health care professionals (nursing and medicine) (n = 12) who were purposely selected to represent a wide range of expertise. Advisory group discussions focused on refining written content, design, tone, graphics, and usability (see Figure 1, Supporting Information). Those that could not attend all meetings were offered the opportunity to provide feedback via email.

Different to those carers who volunteered in the workshop, we also recruited cancer carers to design and develop peer‐led information videos for inclusion in the PLWR. Recruitment of volunteers was conducted via an email distribution to cancer voluntary agencies in the region (n = 20). These charities provide support and advocacy in urban and rural locations for families affected by cancer. Fifteen carers, who cared for a range of cancer patients currently or in the past, volunteered to allow their final interviews to be included in the PLWR. Some carers did not provide consent, and therefore this video material was removed. These 15 carers, guided by the project team, were interviewed by a film director regarding their experiences of caring across the illness journey.

Six cancer‐related professionals (a cancer surgeon; a clinical nurse specialist; a clinical psychologist; a senior social worker; a family cancer service co‐ordinator; and a fitness and wellbeing co‐ordinator) also contributed videoed advice and tips for coping in the caring role.

#### 2.1.2 Step 2: Development of PLWR prototype 1

Information gathered in step 1 was combined and used to develop draft content and PLWR structure. Videos were clipped and themed for inclusion in various sections that mapped the cancer journey, eg, “diagnosis,” “treatment,” and “bereavement.” These trajectory markers were directed by earlier workshop consultations. Development of the PLWR prototype was supported by a film production company and expert web‐designers.

#### 2.1.3 Step 3: User testing phase 1

An unstructured feedback session with six cancer carers who participated in earlier workshops was conducted at the regional cancer centre to gather early views and experiences of users' interactions with the resource. A demonstration of the full PLWR was given. Following the demonstration, cancer carers were encouraged to provide their thoughts on the website and provide suggested changes. These discussions were recorded and themed.

#### 2.1.4 Step 4: Refining PLWR prototype

Changes were made to the resource based on this iterative development. Following agreement with advisory group, these included changes in content, music, and titles of PLWR components (see step 3, Supporting Information).

### 2.2 Phase 2 PLWR user testing

#### 2.2.1 Step 5: User testing and refinement

A different group of carers were recruited to evaluate the PLWR. A multiple‐methods design combining a web‐based survey with an optional semi‐structured telephone interview was used. The survey and interview questions were designed by the advisory team based on earlier research.^9^, ^24^, ^25^ Cancer carers were recruited by OS and TMcS via a range of 25 outpatient cancer clinics (including chemotherapy, radiotherapy, surgery, and follow‐up), over an 8‐week period. Carers were included if they were currently providing informal care for a patient with cancer, over the age of 18 and had access to the internet at home.

Self‐identified cancer carers were given a personal 5‐minute, one‐to‐one demonstration of the PLWR on an iPad by OS and TMcS in the outpatient clinics. Carers were given a promotional bookmark and pen with the website details and asked to review the entire PLWR at home including all videos and written material. Carers who accessed the website were requested to complete the web‐based survey including a demographic questionnaire (age, gender, relationship to patient, patient diagnosis/time spent caring/stage of cancer) and Likert style survey containing 11 items focusing on content, graphics, videos, relevance, and overall look (see Table 1, Supporting Information). Respondents were also asked to complete open‐ended questions which focused on rating the function and usefulness of the website. Additionally, to increase potential feedback on the site, the PLWR was advertised via the university's Twitter and Facebook account. The web‐based survey was facilitated by the Qualtrics software.

Completed surveys were downloaded to SPSS version 22^27^ for analysis using descriptive statistics. Google analytics were also used to measure the number of page visits, visitor journey through the website, referral to the site, access by device type, and location of user. Administrators and study personnel visits were removed from final analysis.

Carers were offered via a link on the PLWR an optional telephone semi‐structured interview with TMcS. This interview focused on the experiences of using the website, usefulness, and ideas for change. Interviews and open‐ended responses were conducted until the point of data saturation.^28^ All interviews were transcribed verbatim and analysed thematically by OS and TMcS.^29^ The level of agreement between the two researchers regarding coding categories and themes was assessed after the analysis of each transcript. Transcripts were searched for data that may have contradicted the emerging themes; no contradictions were found.

#### 2.2.2 Step 6: Develop final PLWR

Carer responses in step 5 were used to identify need for refinement.

## 3 RESULTS

### 3.1 Phase 1: Development of the resource through co‐design with a range of stakeholders

#### 3.1.1 Step 1‐4

The website was created over a period of 10 months (April 2017 ‐ January 2018). The co‐design process highlighted the need for the PLWR to provide information tailored to specific points of the caring trajectory (see web‐based Supporting Information). Delivering information tailored to specific points in the illness trajectory was suggested as a manageable way for carers to absorb information. Furthermore, carers were described as busy and emotionally fatigued. Providing information via peer‐led videos was suggested as an easily accessible format which carers may find less mentally taxing than absorbing large volumes of written material.

Following step 1, a PLWR “Cancer Caring Coping” www.cancercaringcoping.com was developed. The resource was designed to deliver information regarding common emotions and issues associated with being a cancer carer, and top tips on how to cope and manage the caring role including links to other services. The carers indicated that the resource should tailor advice and support to specific points of the caring trajectory.

#### 3.1.2 Step 5: User testing and refinement phase 1

Analysis of group discussion with carers identified that music included in peer‐led videos was perceived as depressing. Carers also suggested that the PLWR section names which were originally “diagnosis,” “treatment,” and “palliative care” should be modified to the following: “when cancer first came into our lives,” “getting through the treatment,” and “caring for yourself”.

#### 3.1.3 Step 6

Refinements identified in step 5 were made and description of the final resource (see Table 2 Supporting Information).

### 3.2 Phase 2: PLWR user testing

#### 3.2.1 Step 6

##### Google analytics

From 1 November 2017 to 28th May 2018, there were 2769 unique visits to the website with 743 returning visitors (average time 01:58 minutes) with 54% returning twice and 30% making up to 20 visits (see Figure 2, Supporting Information). Peer‐led videos were the most frequently accessed components (see Supporting Information) with carers watching; the full personal stories (414 views, avg time 5.18 minutes), followed by the themed videos: “when cancer first came into our lives” (159 views, average time 2.20 minutes), “getting through the treatment” (121 views, average time spent 2.52 minutes), and “bereavement” (120 views, average time spent 1.78). Carers appeared to make significantly less visits to the professional led material; however, when accessed, they spent extended periods of time on these professional pages, eg, “top tips from nurse” (51 views, average time spent 3.53 minutes).

Content focusing on supporting children (10 views, average time 2.00 minutes) and the emotional aspects of caring (17 visits, average 2.51 minutes average) were less frequently visited. Of the 2769 visits, 1600 visitors accessed multiple components of the resource with the first point of information accessed mainly “getting through treatment” information, “caring for you,” and “financial employment” information before accessing other components.

The most common referral pathway for visitors to the site was Facebook (45%, n = 829), Google (32%, n = 598), and direct (from the link provided) (15%, n = 268). The majority of visitors accessed the website via a mobile device (60%, n = 1112).

##### Web‐based survey responses

Of the 55 who completed the evaluation, 54 rated all features as excellent or good, with only one carer rating the relevance of the website as poor. Cancer carers reported being highly satisfied with the resource and viewed it as useful and relevant.

Thematic analysis of the 54 open‐ended responses and 10 telephone interviews identified four key themes: meeting information needs; peer delivery of information; web‐based delivery of information; and negative experience of the resource.

##### Meeting unmet information needs

Carers perceived the website as a valuable resource due to the current lack of tailored information available. They noted that they often felt ignored within the health care system and that this resource helped to reduce this feeling of isolation. Carers expressed that prior to accessing the website they felt ill‐prepared for caring and that HCP time was focused on preparing patients. They reported that the website offered the necessary information needs that in turn reduced stress.

One caregiver summed up their positive reflections on the website:

*‘[I am] very impressed overall. Excellent website, as it focuses entirely on the needs of a carer of someone with cancer. It's important to get the word out there about its existence’* [ Carer, open ended response to survey].

##### Peer‐led delivery

Hearing others report similar emotions and experiences in peer‐led videos was reported to reduce feelings of helplessness and uncertainty surrounding the caregiving role. Carers reported that they found the experiences of peers motivating and inspiring. The use of peer‐led videos was particularly helpful in providing emotional and practical support as the carers felt that the tips and techniques could easily be implemented in day to day life.

The use of real life experiences was described by several carers as desolating and reassuring which in turn helped carers to feel less alone.

*‘Sometimes I feel so alone and confused. I feel helpless. It really helped hearing that I'm not alone and that other people feel the same’* [ Carer, telephone interview].

##### Web‐based delivery

Carers reported that absorbing information in written form whilst caring for someone who has cancer as very challenging due to limited personal time and high emotions which can make it difficult to concentrate to read. Providing videoed information was viewed as convenient as and less burdensome than reading. Carers reported that the web‐based format allowed them to receive tips and advice in a fast and convenient way. This was viewed to be beneficial, particularly those who lived in rural areas.

Carers reported that the website was easy to use due to the clear layout and straightforward access to relevant information.

The themed sections also appealed to carers, providing ease of access to specific supports without the need to read irrelevant material.

*‘[I liked the] easy access to information … It was set out in sections that allows everyone to reference depending on their caring situation’* [ Carer, telephone interview].

##### Negative experiences/potential improvements

Three people reported negative experiences of using the website. One person stated they found that listening to other people's experiences lowered their mood. Two reported that they felt the resource did not represent male carers or families with younger children.

They reported that the website would benefit from an interactive function to facilitate and moderate questions, answers, debates, and general communication. Carers expressed that having the opportunity to interact directly with other carers would provide an additional layer of support.

*“Maybe a way to post questions and chat with other people who have a loved one with cancer so that we can support each other”* [ Carer, telephone interviews].

## 4 DISCUSSION

The international cancer carer literature recognises the need to support carers, but few interventions have been rigorously developed and evaluated.^14^, ^19^ To address this concern, this PLWR was co‐designed to ensure it provided relevant and appropriate information and support to cancer carers. Co‐design identified that the PLWR should include information regarding common emotions, issues, and tips and advice on how to cope with caring. Information was delivered via peer‐led videos with accompanying written information and signposting to services tailored across the caring trajectory. Iterative co‐design allowed for the early identification of problems and necessary refinements.

Google analytics demonstrated that demand for the resource was good with 2800 visits within 6 months and 750 repeat visits, of these repeat visitors 70% visited the site multiple times. Set in the context of 9000 new cancer cases per year in Northern Ireland,^30^ a high usage trend supports the evidence that carers regularly access web‐based information.^20^, ^31^ The majority of visitors were directed to the site via Facebook and Google and accessed via a mobile device. Wide implementation of web‐based resources for caregivers should focus resource advertising in these arenas and ensure that web‐based interventions are mobile enabled.

In terms of content, site visitors made significantly more visits to the peer‐led aspects compared with the professional‐led content. However, when accessed, HCP content was viewed for prolonged periods (approx. 4 minutes). Despite a higher number of visits to the peer‐led components, finance and employment information was the most commonly first viewed component, followed by the themed carer stories of “treatment” and “caring for you” information, suggesting these as a potential support priority for carers. This supports earlier studies which suggest that finance is often an unmet need for caregivers.^31^, ^32^, ^33^

Carers reported that the resource provided information which helped to normalise emotions and what to expect in the caring role. This social comparison allowed carers to receive information and affiliation with those carers who had survived the experience thus gaining confidence.^34^ The positive response to the resource suggests support for the use of the six‐step co‐design as a mechanism to identify and implement improvements in health care, supporting the view that users' experiences can be improved by listening to the views and opinions of those who use the service.

Carers reported strong acceptability for peer‐led videos reporting that the PLWR reduced isolation, provided emotional support, and reduced uncertainty. This is reflective of the peer‐led literature which demonstrates that participants are highly accepting of peer support, reporting high levels of satisfaction, shared experience, and empathic understanding.^35^, ^36^ Peer narratives can allow experiences to be normalised by developing understanding and meaning, reducing uncertainty and promoting coping.^37^, ^38^ The web‐based format was viewed positively due to the ease of access and no geographic or time barriers. A recent review of web‐based interventions for cancer carers suggested that web‐based interventions can improve coping, psychological wellbeing, burden, and perceived bonding between carers and patients; however, many of these studies have methodological issues^17^ such as small sample size. Further high quality intervention studies are required to rigorously test the effectiveness of web‐based interventions for cancer carers.

### 4.1 Study limitations

Fifty‐five carers participated in user testing of the resource which is a small proportion of the visitors to the site. Potentially carers that viewed the resource negatively were less likely to participate in the evaluation, with responders feeling obliged to comment in a socially desirable way. To overcome this limitation, future studies should consider a purposive sample where carers are asked to instantly discuss their views to someone outside the research team using a think aloud technique.^39^ Other limitations include: the sample of carers participating in the resource evaluation was also homogeneous in terms of age and gender and cancer site; therefore, we cannot guarantee that the resource is acceptable to diverse and minority carers. However, generalisation is likely due to the wide and varied types of carers involved in the development of the PLWR.

### 4.2 Clinical implications

Ongoing evaluation is necessary in order to provide definitive evidence whether the PLWR provides benefits for cancer carers. The PLWR will continue to be implemented regionally with ongoing evaluation. Given the relatively small cost associated with developing the website (£25 000) set against the number of carers who can access the resource, and limited costs associated with updating, the PLWR has the potential to demonstrate positive outcomes for carers in a cost‐effective way.

An e‐platform promotes sustainability and future‐proofing. However, one of the main challenges facing web‐based resources is the ongoing responsibility and funding required to maintain the PLWR. The co‐design approach in development has ensured agreement between the voluntary and statutory sector to make available a link to the resource at a regional level.

The co‐design process was complex resulting in a series of iterative changes and improvements; future research should measure the impact and outcomes associated with co‐design.^17^

The PLWR could easily be implemented into routine clinical practice across numerous countries. Furthermore, the intervention model may be relevant to develop and test with carers of other conditions. Currently, the CCC is being adapted for use in Vietnam, and we have plans to continue to make culturally specific adaptions to meet the needs of cancer carers internationally. In addition, the six‐step co‐design model developed by the team is an effective way to develop appropriate information for service users and could be utilised as a framework for development of other interventions in a variety of disease groups.

## 5 CONCLUSION

Through the process of co‐design, we developed a web‐based resource targeted to cancer carers. We demonstrated the PLWR appears acceptable to carers. Ongoing research is required to test the effectiveness of the resource on caregiver outcomes. There is the potential to extend CCC beyond the regional resource, and progress is already being made towards an internationally relevant web‐based resource for cancer carers. The co‐design model should be used in future service user intervention development to ensure appropriate, relevant content that is accessible by all stakeholders.

## CONFLICT OF INTEREST

None.

## ETHICAL APPROVAL STATEMENT

Reviewed by Queen's University Governance Procedures and Belfast Health and Social Care Trust. Full Ethical approval was not required as project was categorised as Public and Patient Involvement and Service Evaluation.

## Supporting information

Data S1. Supporting Information
