# Supplementary material for: Using a six‐step co‐design model to develop and test a peer‐led web‐based resource (PLWR) to support informal carers of cancer patients
Source: Psychooncology. 2019 Jan 16;28(3):518–24. doi: 10.1002/pon.4969 (PMC6590360; doi:10.1002/pon.4969)
Supplement: Supplementary file 1 — Data S1. Supporting Information [file PON-28-518-s001.docx]

**
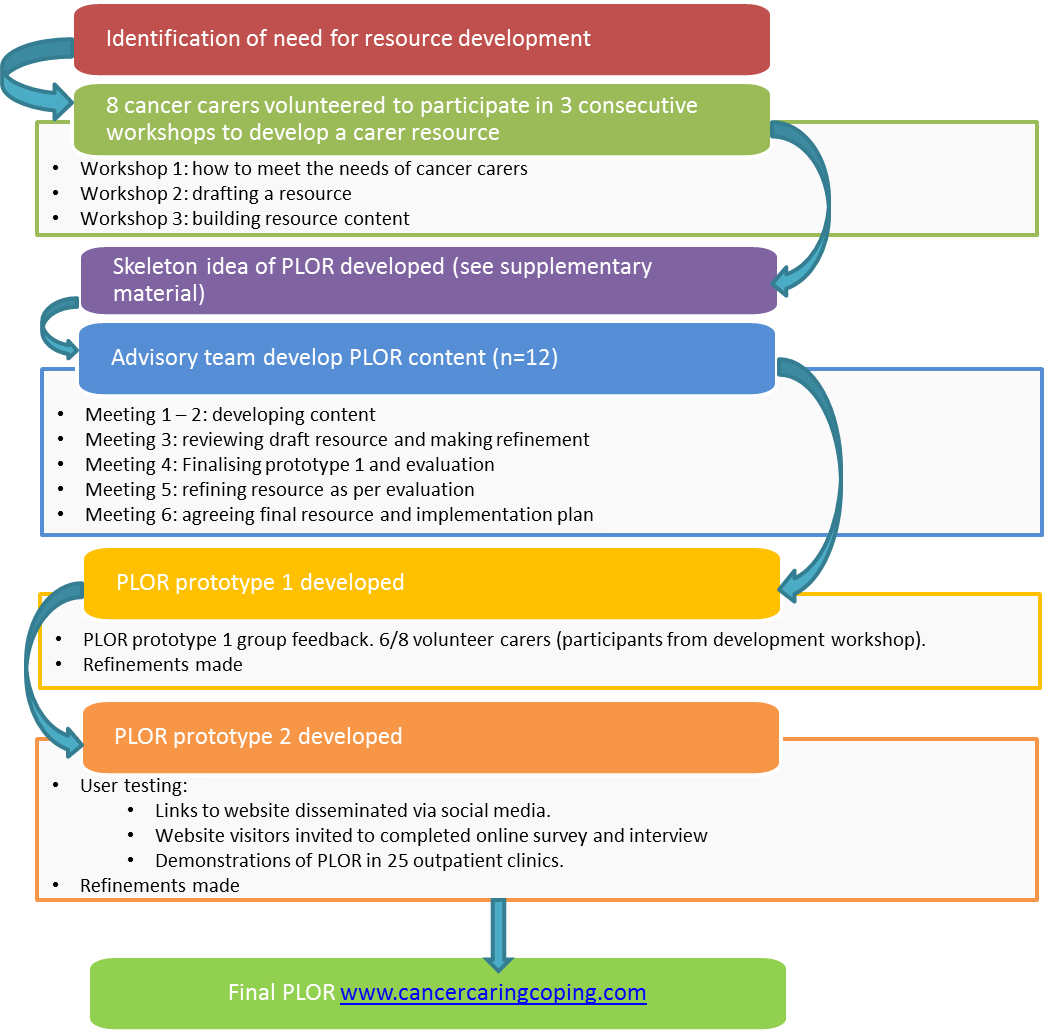
**

**Fig 1**

**Step 1: Workshops Topics Discussed Consumer– User Needs Analysis –developing the content**

*Workshop 1*

Background to the Project – Population based study identified need for information on managing medical issues, psychological issues, financial concerns and other carer’s stories.

- Main issues and concerns of carers
- What supports are available? What supports are required?

*Workshop 2 – Building the online resource*

Advantages/disadvantages of an e-learning resource.

What should be the main aim of the site?

What should the video resource look like? E.g. who should deliver the information/ in what way?

Results of the population based study suggested that carers require information regarding medical issues / psychological concerns / financial issues / carers stories. Are there any other needs that are important to include?

*Workshop 3- Building the content*

What should the website look like? What information should it include?

How technically proficient are you- would you need help or assistance? What help?

Do you use any similar sites? What is good / bad about them?

Content Medical information? What types of medical/ self-management information do carers require? Who should deliver this information, how should the information be delivered?

Psychological/ coping information? What psychological information / resources do people require?

**STEP1 : Description of workshop themes**

**Vehicle for knowledge exchange**

A web site is proposed which would be aimed at individuals who care for loved ones with Cancer– be it a partner, sibling, parent, where often their own physical and mental health becomes secondary to that of the patient. The site will provide a bank of carer’s experiences, practical information and advice on things that carers often struggle with, but don’t seek help with, as they feel the focus of support should be on the patient’s treatment. This unique resource will contain advice on how to care for their own wellbeing and how to deal with other social issues affecting families who are caring for someone with Cancer.

**Web Resource**

The web resource will be user friendly and modular in structure dealing with topics such as diagnosis, living with Cancer, and end of life care. The website will be content manged thus allowing for continuous updates by the project team and partners aiding its sustainability. Headings and themes derived from previous research Santin (2014, 2015). Each section will include short narratives and written text with various types of carers to allow the viewer to select the video that is most suited to them.

It will include multiple video sequences which will provide advice from other carers, and health care professionals on a range of issues. These could include - looking after yourself physically, depression, living on a reduced income, and benefits advice.

The tone of the resource is critical – it must be empathetic, designed by carers for carers to help, guide and support them in their situation. It will provide links to other sources of support such as MacMillan, Citizens Advice Bureau and Cancer Care.

Audience Reach

A web site has the potential to reach the widest audience – of all ages. It will have a simple, uncluttered look and feel which allows the user to navigate to the area they are interested in and to dip in and out the resource as required. It is also possible to include interactive activities or quizzes. This is particularly important to engage a male audience.

It will also contain a number of downloadable PDF’s to allow users the option for printing a hard copy as well as selection for a hard copy DVD. Simple google analytics will be used to monitor traffic to the site and evaluate the output.

**Step 3: Resource Changes**

**Change 1: Changes to the section titles**

Diagnosis change to – When Cancer First Came Into Our Lives

Treatment change to – Getting through the treatment

Bereavement – Bereavement and Life After

**Changes 2: Videos**

Collate all professional videos in a section titled ‘Top tips from professionals’.

Additional video to be added focused on caring for yourself

Music changes in videos- more upbeat

Add summary of information as top tips in each section

**General Changes**

Add short descriptors in each section

Change general background colour from red to teal

Make text bigger/ easier to read

Add a get in touch button

Create a useful links section

**Table 1 Likert Scale**

|  | Excellent | Good | Poor | Terrible |
| --- | --- | --- | --- | --- |
| Logo | 4 | 3 | 2 | 1 |
| Name | 4 | 3 | 2 | 1 |
| Fonts | 4 | 3 | 2 | 1 |
| Pictures | 4 | 3 | 2 | 1 |
| Videos | 4 | 3 | 2 | 1 |
| Tips | 4 | 3 | 2 | 1 |
| Links | 4 | 3 | 2 | 1 |
| Menu | 4 | 3 | 2 | 1 |
| Navigation | 4 | 3 | 2 | 1 |
| Relevance | 4 | 3 | 2 | 1 |

Table 2: Description of PLOR sections and content

| Section | Video Content |
| --- | --- |
| When cancer first came into our lives | Carer led videos  Common feelings/emotions. Advice on how to manage: appointments, communication with professionals and family and shock. |
| Getting through the treatment: | Carer led  Managing and coping with treatment days; managing sickness, eating, medications, communication and self-care. |
| Caring for yourself: | Carer led  Importance of self-care, holistic therapies, coping mechanisms, healthy lifestyle. |
| Supporting you to care: | Carer led  Accessing help, providing space and time, family conflict, normal feelings, reflection and managing change, preparing for death and dying |
| Bereavement and life after caring: | Carer led  Feelings and emotions, what to expect, managing the grief of others. Supports, getting back to routine. |
| Life after cancer | Carer led  Managing recovery, lifestyle changes, making positive changes, making friends and connections. |
| Financial and employment support: | Social worker led  Finance, employment, insurance , travelling and car parking assistance, financial benefits, financial advisors, returning to work, supporting parents, and occupational pensions. |
| Emotional Support: | Psychologist led  Emotions, accessing the supports around you, using hobbies and activities, managing anxiety and fear, focusing on the here and now. |
| Supporting Children | Family service co-ordinator led  Talking to children about cancer and its treatment  Managing change when children are involved  Managing school and activities  Sources of support |
| Top tips for caring | Cancer specialist led  Attending and managing medical consultations  How to access support  Communication  Prioritising tasks |

Table 3 showing PLOR visits and visit duration

|  | Page | Page views | Avg.Time on Page (Mins) |
| --- | --- | --- | --- |
| 2 | Our Stories | 414 |  |
| 3 | When Cancer First Came Into Our Lives | 159 | 5.18 |
| 4 | Getting Through The Treatment | 121 | 2.20 |
| 5 | Bereavement and Life After Caring | 120 | 2.52 |
| 6 | Caring for Yourself | 120 | 1.78 |
| 7 | Supporting You to Care | 95 | 2.28 |
| 8 | Top Tips from Professionals | 62 | 1.93 |
| 9 | Financial and Employment Support | 61 | 2.89 |
| 10 | Top Tips from Nurse | 51 | 3.53 |
| 11 | Emotional Impact of Caring | 36 | 1.25 |
| 12 | Top Tips From Surgeon | 29 | 2.58 |
| 13 | Support for the Emotional Impact of Caring | 17 | 2.51 |
| 14 | Supporting Children | 10 | 2.00 |

Fig 2: Number of sessions per returning visitors

**Number of sessions**

%
